# Macrophages reprogram after ischemic stroke and promote efferocytosis and inflammation resolution in the mouse brain

**DOI:** 10.1111/cns.13256

**Published:** 2019-11-07

**Authors:** Wenting Zhang, Jingyan Zhao, Rongrong Wang, Ming Jiang, Qing Ye, Amanda D. Smith, Jun Chen, Yejie Shi

**Affiliations:** ^1^ Department of Neurology Pittsburgh Institute of Brain Disorders & Recovery University of Pittsburgh Pittsburgh PA USA; ^2^ Geriatric Research, Education and Clinical Center Veterans Affairs Pittsburgh Health Care System Pittsburgh PA USA

**Keywords:** apoptotic cell clearance, focal cerebral ischemia, inflammation resolution, phagocytosis, RNA‐seq

## Abstract

**Aims:**

Blood‐borne monocytes/macrophages infiltrate the brain in massive numbers after ischemic stroke, but their impact on poststroke brain injury and recovery remains elusive. This study examined the transcriptomic changes in monocytes/macrophages after ischemic stroke and the functional implications of these changes, particularly with regards to the contribution of these cells to the phagocytic clearance of dead/dying cells (efferocytosis) in the poststroke brain.

**Methods:**

We performed whole‐genome RNA sequencing on the monocyte/macrophage population sorted from mouse brain and peripheral blood 5 days after permanent focal cerebral ischemia. In addition, the spatial and temporal profiles of macrophage efferocytosis were examined in vivo by immunohistochemistry 3‐7 days after brain ischemia.

**Results:**

Robust transcriptomic changes occurred in monocytes/macrophages upon infiltrating the poststroke brain. Functional enrichment analysis revealed a transcriptome of brain macrophages that strongly favored efferocytic activity. A large number of efferocytosis‐related genes were upregulated in brain macrophages, the products of which are essential components involved in various steps of efferocytosis, such as chemotaxis, recognition of dead cells, engulfment, and processing of phagosomes. The efferocytic activity of brain macrophages were verified by immunohistochemistry, wherein Iba1‐labeled microglia/macrophages effectively cleared apoptotic neurons in the infarct during the subacute stage after brain ischemia. We also identified PPARγ and STAT6 as potential upstream regulators that shaped this proefferocytic and inflammation‐resolving transcriptome of macrophages in the poststroke brain.

**Conclusion:**

Macrophages play a crucial role in the phagocytic clearance of dead neurons after ischemic stroke and promote the resolution of inflammation in the brain. Molecular therapies that enhance macrophage efferocytic capability may be promising treatments for ischemic stroke by facilitating inflammation resolution, brain repair, and recovery of neurological functions.

## 1 INTRODUCTION

Ischemic stroke is a devastating condition for which there is currently no effective therapy against its neurological sequelae. When ischemic stroke occurs, loss of blood supply causes neuronal damage in the ischemic core within hours, which can spread to the potentially salvageable penumbra area without intervention.^1^, ^2^ The injured neurons release danger‐associated molecular patterns (DAMPs) to initiate a cascade of inflammatory responses as an endogenous means to limit injury.^3^ However, persistent encounter with dead/dying neurons also leads to excessive inflammation, potentiating secondary brain injury and hindering brain repair.^3^, ^4^ Phagocytic clearance of dead cells, also known as efferocytosis, is an important function of phagocytes to resolve local inflammation and restore function of damaged tissues.^5^ Prompt disposal of dead cells and debris also fosters a permissive local milieu that facilitates tissue regeneration. To date, the cellular and molecular mechanisms underlying efferocytosis in the brain after ischemic stroke, and its impact on brain injury and recovery, are poorly understood.

Ischemic stroke induces massive infiltration of blood‐borne immune cells into the brain, which are recruited to the site of injury by a variety of chemokines.^3^, ^6^, ^7^, ^8^, ^9^ Monocytes/macrophages are a subset of these immune cells present in high numbers,^10^, ^11^, ^12^, ^13^ but their function in the poststroke brain is not well defined. Macrophages have long been known as professional phagocytes due to their high capacity of engulfment.^5^ In peripheral organs such as the lung,^14^ liver,^15^ and heart,^16^ as well as in in vitro cultures,^17^ macrophages phagocytose dead cells and play an instrumental role in the maintenance of tissue homeostasis, mitigation of inflammation after injury or infection, and promotion of tissue regeneration. However, whether macrophages participate in efferocytosis in the injured CNS after ischemic stroke, and the consequences of efferocytosis with respect to long‐term stroke outcomes have not been thoroughly examined.

In the present study, we investigated genomic changes in monocytes/macrophages invading the brain after ischemic stroke by RNA sequencing (RNA‐seq) and explored the functional implications of any alterations in the macrophage transcriptome. We report that monocytes/macrophages undergo genomic reprogramming in response to ischemic stroke and acquire a transcriptome favoring phagocytic clearance of dead cells in the poststroke brain, thereby promoting inflammation resolution and neurorestoration.

## 2 METHODS

### 2.1 Animals

C57BL/6J mice were purchased from the Jackson Laboratory. Mice were housed in a temperature and humidity‐controlled animal facility with a 12‐hour light/dark cycle with food and water available ad libitum. All animal procedures were approved by the University of Pittsburgh Institutional Animal Care and Use Committee and performed in accordance with the *Guide for the Care and Use of Laboratory Animals*. All efforts were made to minimize animal suffering and the number of animals used.

### 2.2 Permanent focal cerebral ischemia

Focal cerebral ischemia was induced in male mice (8‐12 weeks old, 25‐30 g) using the distal middle cerebral artery occlusion (dMCAO) model, as described previously.^18^ Briefly, a skin incision was made at the midline of the neck, and the left common carotid artery was exposed and ligated. A craniotomy was performed between the left eye and the ear to expose the junction of the rhinal fissure and MCA. The distal branch of the left MCA was then occluded with low intensity bipolar electrocautery at the immediate lateral part of the rhinal fissure. Both the rectal temperature and the left temporalis muscle temperature were maintained at 37.0 ± 0.5°C during surgery with a temperature‐controlled heating pad and a heat lamp. Mean arterial blood pressure was monitored during surgery by a tail cuff. Cortical cerebral blood flow (CBF) was measured using two‐dimensional laser speckle imaging before and after ischemia according to the manufacturer's instructions. Failure to reduce CBF to 30% or less of baseline levels led to subject exclusion (<5%) from further experimentation. Experimental procedures were performed following criteria derived from the Stroke Therapy Academic Industry Roundtable guidelines for preclinical evaluation of stroke therapeutics.^19^ Accordingly, experimental group assignment was randomized with a lottery‐drawing box, and surgeries and all outcome assessments were performed by investigators blinded to group assignment.

### 2.3 Fluorescence‐activated cell sorting

Single‐cell suspensions were prepared from mouse blood and brain as described previously.^20^, ^21^ Briefly, mice were deeply anesthetized and transcardially perfused with sterile 0.9% NaCl. The ipsilesional brain hemisphere was harvested, and cell suspensions were prepared using the Neural Tissue Dissociation Kit and gentleMACS Octo Dissociator with Heaters (Miltenyi Biotec) following the manufacturer's instructions. Suspensions were passed through a 70‐μm cell strainer, and fractionated on a 30% and 70% Percoll gradient at 500 g for 30 minutes. Peripheral blood was obtained from mice by cardiac puncture and fractioned in Ficoll solution at 500 g for 15 minutes. Mononuclear cells at the interface were collected, resuspended at 1 × 10^6^/mL, and stained with the following fluorophore‐conjugated antibodies and the appropriate isotype controls: anti‐CD45 (eBioscience 45‐0451‐82), anti‐CD11b (eBioscience 17‐0112‐83), anti‐Ly6G (eBioscience 11‐9668‐82), and anti‐CD11c (BD Biosciences 562782). Fluorescence‐activated cell sorting (FACS) was performed using the FACS Aria I sorter and FACS Diva software (BD Biosciences). Monocytes and macrophages were gated as CD11c^−^Ly6G^−^CD11b^+^CD45^high^ and sorted for bulk RNA‐seq. For blood monocytes, each sample was extracted from one mouse. For brain macrophages, each sample was pooled from five mouse brains to obtain enough cells. Three samples (biological replicates) for each experimental group were collected for RNA sequencing.

### 2.4 RNA sequencing

RNA extraction, library preparation, and sequencing were performed at the University of Pittsburgh HSCRF Genomics Research Core. Briefly, total RNA was extracted from FACS‐sorted cells using RNeasy Plus Micro Kit (Qiagen). RNA integrity was assessed using the High Sensitivity RNA ScreenTape system on an Agilent 2200 TapeStation (Agilent). The SMART‐Seq HT Kit (Takara Bio) was used to generate cDNA from 10 ng of total RNA, and the cDNA product was checked by an Agilent Fragment Analyzer system (Agilent) for quality control. The sequencing library was constructed by following the Illumina Nextera XT Sample Preparation Guide. One nanogram of input cDNA was tagmented and amplified using the Illumina Nextera XT kit. Equimolar amounts of each sample were finally pooled and sequenced on an Illumina Nextseq 500 system, using a paired‐end 75‐bp strategy.

### 2.5 RNA‐seq data analysis

All RNA‐seq data are deposited in the Gene Expression Omnibus database at the National Center for Biotechnology Information (GSE138805). Preprocessing of the RNA‐seq data was completed using Chipster.^22^ Fastq files were quality controlled using FastQC,^23^ and all samples passed quality control criteria. Reads were mapped to the GRCm38 mouse genome using HISAT2^24^ and counted by HTSeq^25^ Genes were identified by Ensembl ID.^26^ The R package DESeq2^27^ was used to normalize the counts and to perform differential expression analysis. Differentially expressed genes (DEGs) were defined as genes with a fold change >2 or <−2, and with a Benjamini‐Hochberg adjusted *P*‐value <.05. Normalized read counts were used for gene expression profiling and were log_2_(*x* + 1)‐transformed. For principal component analysis (PCA), variance‐stabilizing transformation was performed on normalized counts for each sample. Heat maps were generated using the R package *pheatmap*. Normalized counts were log transformed, *z*‐scaled, and used as input data for heat map construction.

Differentially expressed genes identified by DESeq2 were submitted to Ingenuity Pathway Analysis (IPA) for pathway analysis using the Ingenuity Knowledge Base (Qiagen Bioinformatics). The fold change and adjusted *P*‐value for each gene were used to perform the core analysis. Diseases and functions were considered significantly enriched with a *P*‐value of overlap <.01 and an activation *z*‐score >2 (predicted to be activated) or <−2 (predicted to be inhibited). The *Upstream Regulator* analysis was used to identify the cascade of upstream transcriptional regulators that can explain the observed gene expression changes in the dataset. An upstream regulator was predicted to be strongly activated or inhibited if its activation *z*‐score was >2 or <−2, respectively. The cutoff *P*‐value was set at .01.

### 2.6 Immunohistochemistry and data analyses

Mice were deeply anesthetized and transcardially perfused with 0.9% NaCl, followed by 4% paraformaldehyde in PBS.^28^ Brains were harvested and cryoprotected in 30% sucrose in PBS, and frozen serial coronal brain sections (25‐μm thick) were prepared on a sliding microtome (Microm HM 450; Thermo Scientific). Sections were blocked with 5% donkey serum in PBS for 1 hour, followed by overnight incubation (4°C) with the following primary antibodies: rabbit anti‐NeuN (Millipore, MAB377, 1:500) and goat anti‐Iba1 (Abcam, ab48004, 1:500). After washing, sections were incubated for 1 hour at 20°C with donkey secondary antibodies conjugated with DyLight 488 or Cy3 fluorophores (1:1000; Jackson ImmunoResearch Laboratories). To visualize apoptotic cells,^29^ TUNEL staining was performed using an In Situ Cell Death Detection Kit (Roche, 11684795910) following manufacturer's instructions. Brain sections were then mounted and coverslipped with Fluoromount‐G containing DAPI (Southern Biotech).

*Z*‐stack images were taken with an Olympus Fluoview FV1000 confocal microscope and FV10‐ASW 2.0 software. A total of 10 consecutive images with the interspace of 1 μm and total volume of 4.49 × 10^5^ µm^3^ were captured for each microscopic field. Phagocytosis of dead/dying neurons by microglia/macrophages was examined on NeuN/Iba1 double‐labeled or NeuN/Iba1/TUNEL triple‐labeled images in brain regions surrounding the infarct. We first determined the infarct boundary according to the dramatic decrease in the NeuN immunosignal. The proximal area was then defined as the area approximately 200 μm lateral from the infarct border, and the distal area was defined as the area approximately 200 μm lateral from the proximal area. Inner peri‐infarct area was defined as the area inside the infarct and within approximately 100 μm from the infarct border. The rest of the area inside the infarct border was defined as infarct core, characterized by massive accumulation of TUNEL^+^ cells. The numbers of NeuN^+^, Iba1^+^, NeuN^+^Iba1^+^, and NeuN^+^TUNEL^+^Iba1^+^ cells were counted in the aforementioned areas 3, 5, and 7 days after dMCAO by a blinded investigator. The corresponding areas in the nonischemic contralateral hemisphere 5 days after dMCAO served as control. Cells were counted from two randomly selected microscopic fields per brain section, and two sections from each brain were stained. Phagocytic index was calculated as the percentage of dead/dying neurons engulfed by phagocytic microglia/macrophages: (number of NeuN^+^TUNEL^+^Iba1^+^ cells/number of NeuN^+^TUNEL^+^ cells) ×100%.

### 2.7 Imaris 3‐D rendering and quantification

The image‐processing software Imaris (Bitplane; v9.3) was used to reconstruct three‐dimensional images of NeuN, TUNEL, and Iba1 fluorescent cells. Confocal image stacks were imported into Imaris, and the *surface* module was used to generate 3D structures of each color channel. Briefly, a region of interest was selected, and the absolute intensity of each source channel was used for reconstruction. Smoothing was set at 0.400 μm for all channels and images. A threshold was set to differentiate the target signal from background, and the same threshold value was used for all groups. Nonspecific signals were then removed, and the 3D‐rendered images were constructed. All images were processed with the same adjustments and parameters. The surface contact area of NeuN and Iba1 immunofluorescence was manually reconstructed and calculated using a Matlab plugin *surface‐surface contact* in Imaris based on the 3D‐rendered images. According to the contact area between NeuN and Iba1 immunosignals, we classified Iba1^+^ cells into four types (Videos [Link], [Link], [Link], [Link]): (a) “No touch,” the Iba1^+^ cell had no contact area with any NeuN^+^ cell in the area examined; (b) “Touch,” the Iba1^+^ cell had a contact area ≤30% of the NeuN^+^ cell surface area; (c) “Enwrap,” the Iba1^+^ cell had a contact area >30% and <100% of the NeuN^+^ cell surface area; and (d) “Engulf,” a NeuN^+^ cell was completely inside an Iba1^+^ cell.

### 2.8 Statistical analysis

High‐throughput sequencing data were analyzed as described above. Other datasets are presented as mean ± SD. Differences in means among multiple groups were analyzed using one or two‐way ANOVA, followed by the Bonferroni/Dunn post hoc correction. A *P*‐value <.05 was deemed statistically significant. All statistics are summarized in Table S1.

## 3 RESULTS

### 3.1 Genomic reprogramming of monocytes/macrophages in the brain after ischemic stroke

**Figure 1:**
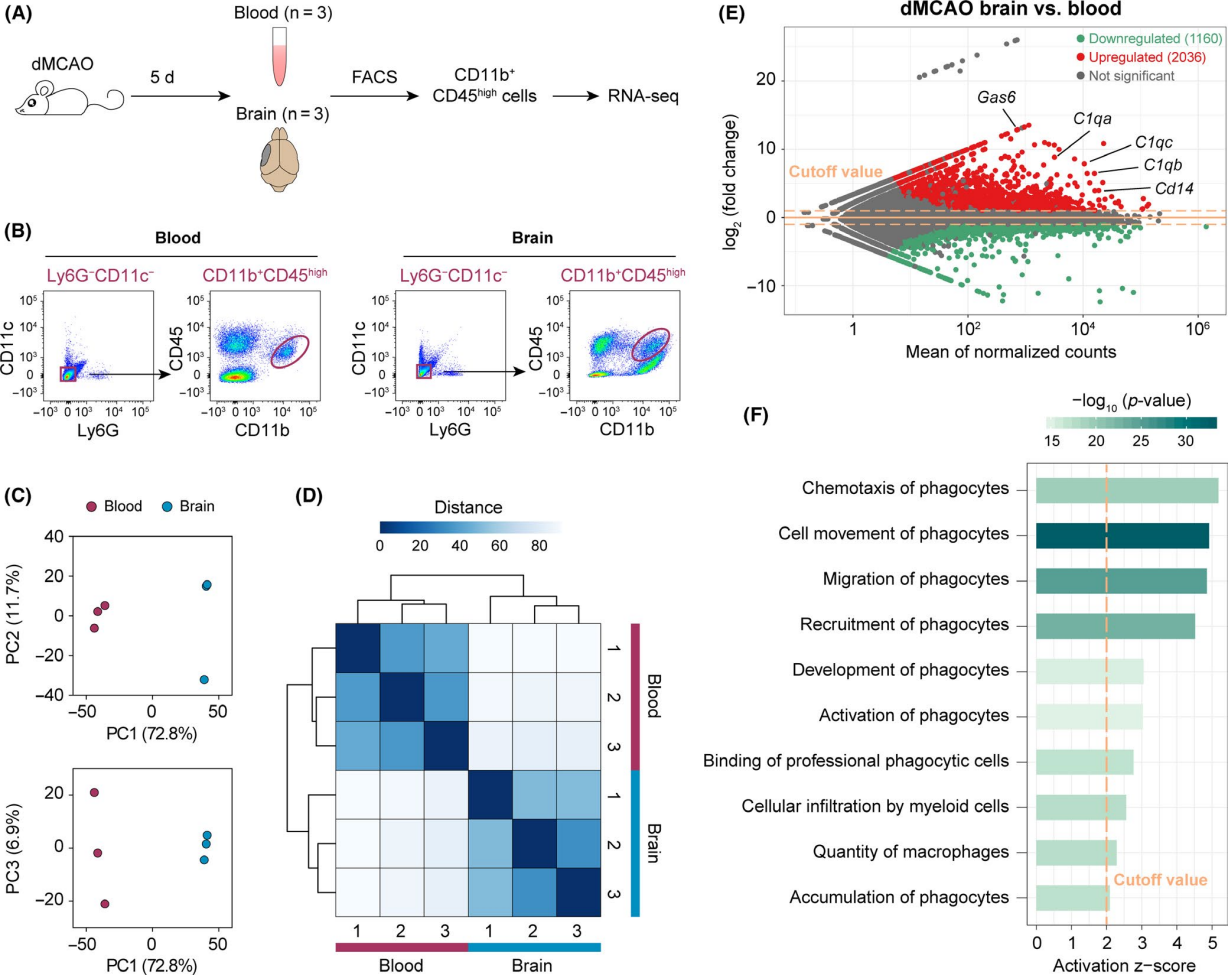
Whole‐genome transcriptomic profiling of monocytes/macrophages in the mouse blood and brain after ischemic stroke. A, Illustration of experimental design. Mice were subjected to permanent focal cerebral ischemia induced by distal middle cerebral artery occlusion (dMCAO). The monocyte/macrophage population (CD11b^+^CD45^high^ cells) were sorted from blood and brain by FACS at 5 days after dMCAO and subjected to RNA‐seq. There were three biological replicates in each group. B, Representative FACS density plots illustrating the cells sorted for RNA‐seq. Cells were gated as Ly6G^−^CD11c^−^CD11b^+^CD45^high^. C, Principal component analysis was performed on RNA‐seq expression profiles of FACS‐sorted cells. Samples in the same experimental group clustered together on principal component 1 (PC1). D, Heatmap of the sample‐to‐sample Euclidean distances illustrating high similarities among the samples in the same group and disparate profiles among samples in different groups. E, MA plot of average RNA‐seq expression differences showing the differentially expressed genes (DEGs; fold change >2 or <−2, adjusted *P*‐value <.05) in monocytes/macrophages from the post‐dMCAO brain versus blood. F, Ingenuity Pathway Analysis (IPA) performed on the DEGs in cells from the brain versus blood predicted several functions of phagocytes to be significantly activated (activation *z*‐score >2, *P*‐value of overlap <.01)

To elucidate the functional roles of monocytes/macrophages in the poststroke brain, the monocyte/macrophage population (CD11c^−^Ly6G^−^CD11b^+^CD45^high^ cells) was sorted from the brain and peripheral blood by FACS 5 days after dMCAO (Figure 1A, B). Sorted cells were subjected to whole‐genome RNA‐seq profiling to investigate the transcriptomic changes of monocytes/macrophages upon entering the poststroke brain. PCA performed on RNA‐seq data demonstrated clustering of samples in the same group and clear separation of samples from different groups on PC1 (Figure 1C), suggesting robust transcriptomic differences between the two experimental groups. Similarly, sample‐to‐sample Euclidean distances calculated on RNA‐seq expression profiles showed high transcriptomic similarity among the three samples in each group (Figure 1D). A total of 3196 genes were differentially expressed between monocytes/macrophages from the brain and blood (fold change >2 or <−2, Benjamini‐Hochberg adjusted *P*‐value <.05), of which 1160 genes were downregulated and 2036 genes were upregulated in post‐dMCAO brain monocytes/macrophages versus blood monocytes (Figure 1E). Notably, several highly variable genes, which had high mean expression levels and large fold changes, were functionally linked to efferocytosis. For example, genes encoding the efferocytic receptor CD14^30^ and several bridging molecules that facilitate the recognition of apoptotic cells (GAS6 and C1q)^30^ were upregulated in brain monocytes/macrophages (Figure 1E), suggesting that monocytes/macrophages in the brain may participate in phagocytic clearance of dead cells after ischemic stroke. Therefore, we assessed the overall functional status of brain monocytes/macrophages by IPA and identified a panel of phagocytic functions that were predicted to be significantly activated (*z*‐score >2, *P*‐value <.01) in brain macrophages, such as chemotaxis, recruitment, activation, and accumulation (Figure 1F).

### 3.2 Brain macrophages upregulate genes involved in the recognition of apoptotic cells after ischemic stroke

**Figure 2:**
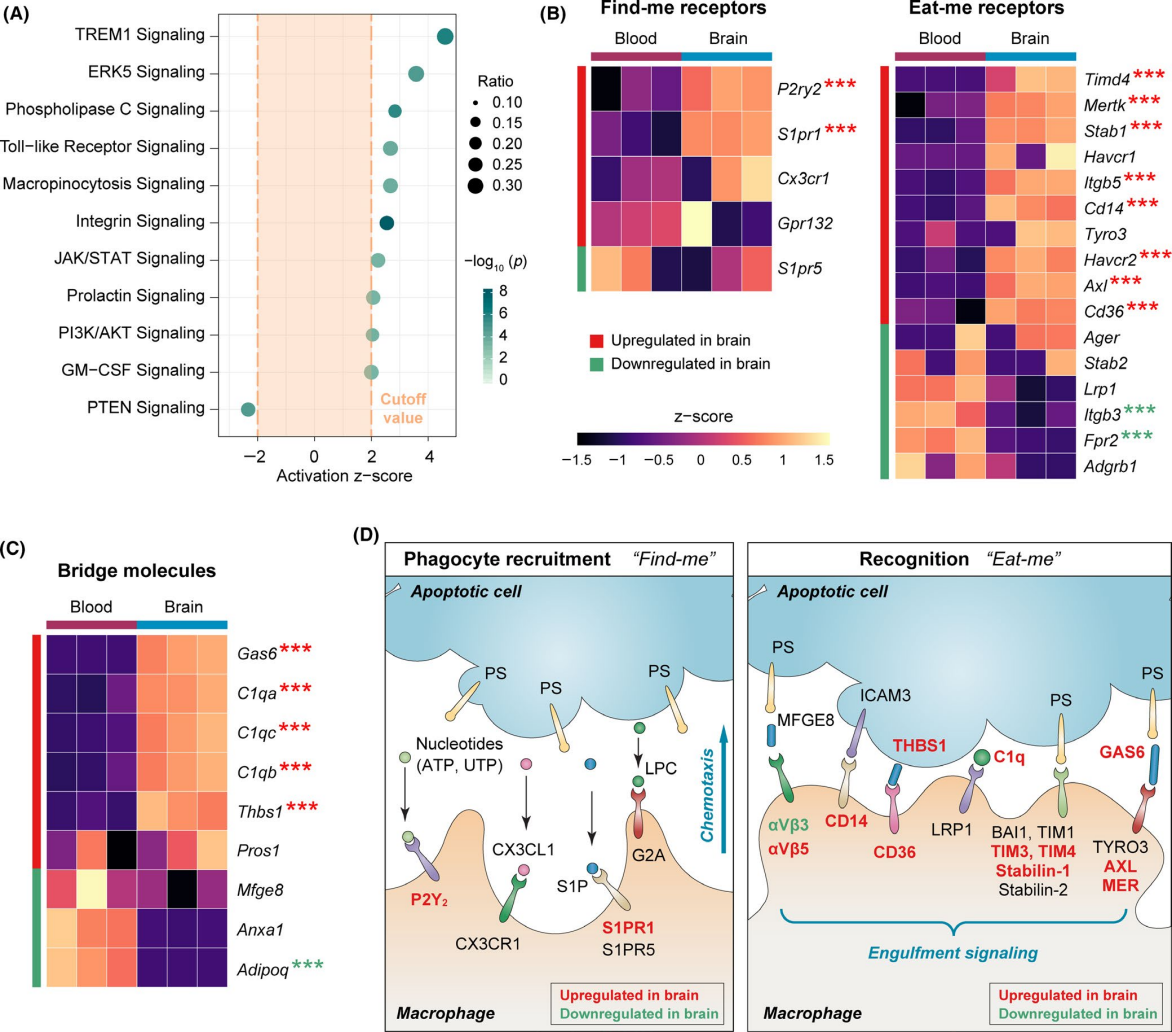
Poststroke brain macrophages upregulate genes participating in the recognition of apoptotic cells during efferocytosis. A, IPA was performed on the DEGs in monocytes/macrophages from post‐dMCAO brain versus blood. Shown are the phagocytosis‐related canonical signaling pathways predicted to be significantly activated (*z*‐score >2, *P* < .01) or inhibited (*z*‐score <−2, *P* < .01) in brain macrophages. B, Heatmaps of the expression profiles of receptors involved in the “find‐me” (left panel) and “eat‐me” (right panel) steps of macrophage efferocytosis. C, Heatmap of the expression profiles of bridge molecules that assist with the recognition of apoptotic cells during macrophage efferocytosis. Each row represents one gene, and each column represents one sample. Color represents the scaled expression value (row *z*‐score). Genes that were significantly upregulated or downregulated in brain macrophages were labeled by asterisks, where *** indicates an adjusted *P*‐value <.001. D, The DEGs in B and C were manually annotated according to their functional roles in the phagocyte recruitment stage (left panel) and recognition stage (right panel) of efferocytosis. Genes that were significantly upregulated or downregulated in brain macrophages were labeled in red or green, respectively. LPC, lysophosphatidylcholine. PS, phosphatidylserine. S1P, sphingosine‐1‐phosphate

We further characterized the monocyte/macrophage transcriptome associated with phagocytic clearance of dead cells at the gene level. First, we used IPA to examine the activation/inhibition status of several canonical signaling pathways known to be involved in phagocytic functions (Figure 2A). A large number of signaling pathways which are known to promote phagocytic activities were predicted to be significantly activated (*z*‐score >2, *P* < .01) in macrophages from the poststroke brain, such as the TREM1 signaling,^31^ ERK5 signaling,^32^ JAK/STAT signaling,^33^ and PI3K/AKT signaling pathways^34^ (Figure 2A). Consistently, the PTEN signaling pathway, which is an endogenous inhibiting signal of phagocytosis,^35^ was predicted to be strongly inhibited (*z*‐score <−2, *P* < .01; Figure 2A) in brain macrophages after cerebral ischemia, further supporting the overall enhanced phagocytic capacity of brain macrophages.

Next, we assessed the expression levels of a panel of receptors that are typically expressed by phagocytes and participate in the clearance of dead/dying cells (Figure 2B). Dying cells can release soluble chemoattractant “find‐me” signals, such as nucleotides (ATP and UTP), sphingosine‐1‐phosphate (S1P), lysophosphatidylchloline, and the chemokine CX3CL1.^36^ On the other hand, the phosphatidylserine (PS) on the outer leaflet of plasma membrane is exposed in apoptotic cells, serving as a critical “eat‐me” signal.^37^ Efferocytosis is initiated when phagocytes are recruited toward the apoptotic cell through binding to the “find‐me” signals and subsequently recognize the “eat‐me” markers on the dying cell.^36^ To this end, genes encoding the purinergic receptor P2Y2 and S1P receptor 1 (S1PR1), two important receptors involved in phagocyte recruitment through binding to nucleotides and S1P, respectively, were significantly upregulated in brain monocytes/macrophages compared with blood monocytes (Figure 2B left panel). Furthermore, many genes encoding receptors essential to recognizing apoptotic cells, either through direct binding to PS or through the assistance of soluble bridging molecules,^36^ were upregulated in brain macrophages (Figure 2B right panel). These included the T‐cell immunoglobulin and mucin‐domain containing and stabilin receptors that directly bind to PS, the TAM receptors Axl and Mer that recognize PS through the bridging of growth arrest‐specific gene 6 (GAS6), αVβ5 integrin that binds to PS through the bridging of milk fat globule EGF factor 8 (MFGE8), CD14, and CD36 (Figure 2B right panel). We also observed upregulation in the expression of several soluble bridging molecules in brain macrophages, such as GAS6, C1q, and THBS1 (Figure 2C). It should be noted that such extracellular molecules are not necessarily secreted by phagocytes themselves. Future studies measuring the protein levels of these soluble factors in the brain are warranted, which considers the contribution from all types of cells in the poststroke brain. In summary (Figure 2D), apoptotic cells can release chemoattractant “find‐me” signals to attract phagocytes to the site of injury and express eat‐me signals to facilitate their recognition through specific receptors on phagocytes. Our data revealed upregulation of a large group of “find‐me” and “eat‐me” receptors in brain macrophages, indicating that these cells are actively engaged in the phagocytic clearance of dead cells during the subacute stage (5 days) after dMCAO.

### 3.3 Poststroke brain macrophages harbor transcriptional changes that favor the engulfment of apoptotic cells

**Figure 3:**
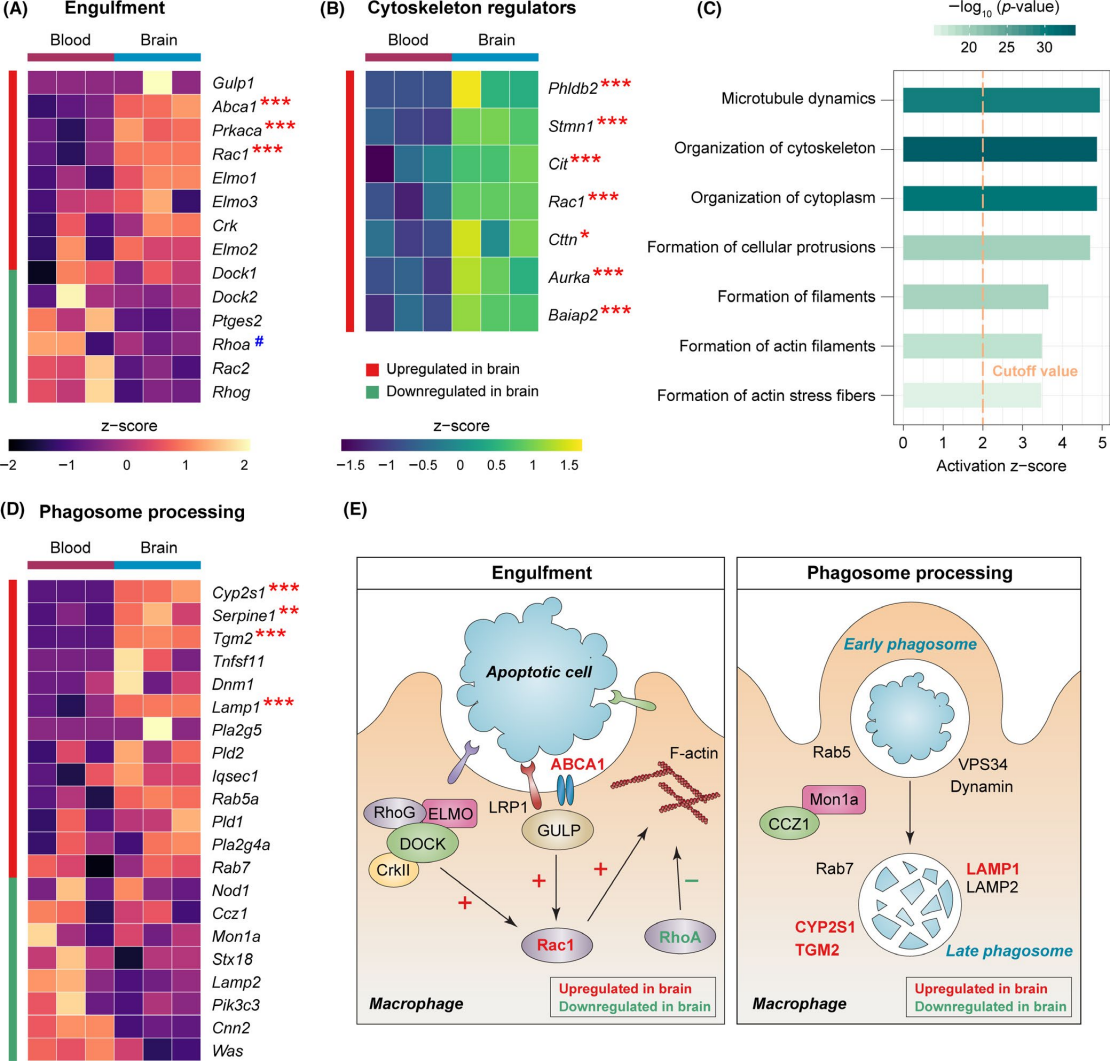
Poststroke brain macrophages harbor transcriptional changes that favor the engulfment of apoptotic cells. A, Heatmap of the expression profiles of cell signaling molecules that regulate the engulfment of apoptotic cells during macrophage efferocytosis. B, Expression profiles of a panel of cytoskeleton regulators, which were upregulated in macrophages from the post‐dMCAO brain versus blood. C, IPA was performed on the DEGs in macrophages from post‐dMCAO brain versus blood. Shown are the functions related to cytoskeleton dynamics predicted to be significantly activated (*z*‐score >2, *P* < .01) or inhibited (*z*‐score <−2, *P* < .01) in brain macrophages. D, Heatmap of the expression profiles of molecules that regulate the processing of phagosomes during efferocytosis. Asterisks label the significantly upregulated (red) or downregulated (green) genes in brain macrophages, where *, **, and *** indicate an adjusted *P*‐value <.05, .01, and .001, respectively. # indicates that the gene product typically inhibits engulfment signaling. E, Genes participating in the engulfment of apoptotic cells (left panel) and subsequent phagosome processing (right panel) were manually annotated. Genes that were significantly upregulated or downregulated in brain macrophages were labeled in red or green, respectively

Once the apoptotic cell is recognized by phagocytes, the binding between ligand and efferocytic receptors triggers a cascade of intracellular signaling that leads to the engulfment of apoptotic cells.^30^ Such signaling involves the engagement of two major complexes, CrkII/ELMO/DOCK and GULP/ABCA1, both of which result in the activation of the small Rho GTPase Rac1.^30^ Rac1 and its countermeasure RhoA exert antagonistic roles in modulating cytoskeletal rearrangement, a prerequisite for phagocytic engulfment of apoptotic cells.^38^ In our dataset, *Abca1* was upregulated in brain macrophages, whereas the levels of *Crk*, *Elmo1*, *Elmo2*, *Elmo3*, *Dock1*, *Dock2*, and *Gulp1* were not different between brain macrophages and blood monocytes (Figure 3A). Nevertheless, Rac1 was significantly upregulated in brain macrophages and mean expression level of RhoA was decreased in brain macrophages, although this decrease was not significant (*P* = .1). Since Rac1 enhances the rearrangement of cytoskeleton and RhoA inhibits this process,^38^ these data suggest that active cytoskeletal remodeling may occur in brain macrophages. Indeed, a panel of seven cytoskeletal regulators was upregulated in brain macrophages, including *Phldb2*, *Stmn1*, *Cit*, *Rac1*, *Cttn*, *Aurka*, and *Baiap2* (Figure 3B). On a functional level, IPA also predicted strong activation of biological processes related to cytoskeletal remodeling in brain macrophages, such as organization of cytoskeleton, organization of cytoplasm, formation of cellular protrusions, and formation of actin filaments (Figure 3C).

After engulfment, the phagosome is formed in the cytosol of phagocytes to facilitate the degradation of cellular debris. We examined a panel of 21 markers known to be involved in phagosome processing and identified four markers that were significantly upregulated in brain macrophages, that is, *Cyp2s1*, *Serpine 1*, *Tgm2*, and *Lamp1* (Figure 3D). The majority of markers examined, however, were not different between brain macrophages and blood monocytes in our dataset (Figure 3D), possibly implying relatively little phagosome processing activity at this stage (5 days) after dMCAO. In summary (Figure 3E), our data support active engulfment signaling and cytoskeletal arrangement in brain macrophages, but phagosome processing was relatively limited. Since our RNA‐seq samples were collected 5 days after dMCAO, this pattern could reflect a snapshot of the dynamic evolution of efferocytic functions at the subacute stage after brain ischemia. More phagosome processing activities in macrophages may be observed at a later stage after dMCAO.

### 3.4 Activated microglia and macrophages phagocytose dead/dying neurons at the subacute stage after cerebral ischemia

**Figure 4:**
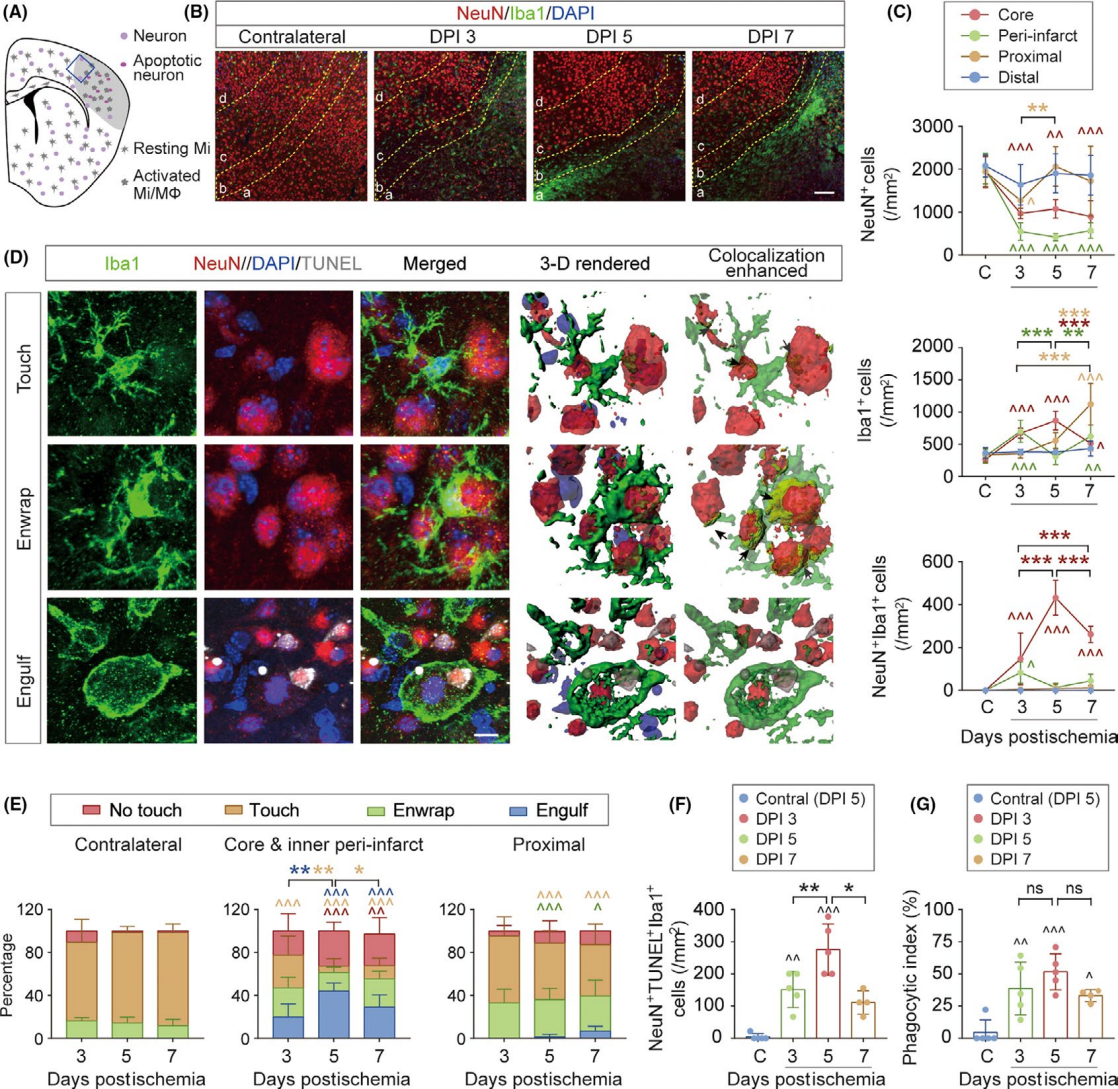
Activated microglia and macrophages phagocytose dead/dying neurons at the subacute stage after cerebral ischemia. Mice were subjected to permanent focal cerebral ischemia induced by dMCAO. A, A schematic cartoon illustrating the distribution of morphologically viable neurons, dead/dying neurons, resting microglia (Mi), and activated microglia/macrophages (Mi/MΦ) in the ipsilesional hemisphere during the subacute stage (3‐7 days) after dMCAO. Gray area: infarct. Blue square: the area surrounding the infarct where images in B were taken. B, Representative images of NeuN, Iba1, and DAPI triple‐label fluorescence in the ipsilesional hemisphere 3, 5, and 7 days postischemia (DPI) and in the noninjured contralateral hemisphere 5 DPI. The image field was divided into four areas as described in Section 2: (a) infarct core; (b) inner peri‐infarct area; (c) proximal area; and (d) distal area. Scale bar: 100 μm. C, Quantification of NeuN‐positive cells (top), Iba1‐positive cells (middle), and NeuN/Iba1 double‐positive cells (bottom) in the infarct core, inner peri‐infarct area, proximal area, and distal area at indicated time points after dMCAO, and in the noninjured contralateral hemisphere (“C”) 5 days after dMCAO. D, Representative images of brain sections stained with NeuN, Iba1, DAPI, and TUNEL demonstrating the interactions between neurons and microglia/macrophages 5 days after dMCAO. Color‐merged images were 3D‐rendered by Imaris software (4th column), with NeuN/Iba1 contact surface enhanced (5th column). Arrow: NeuN and Iba1 contact surface (yellow). Iba1^+^ cells were classified into four types according to their contact area with NeuN immunosignal as described in Section 2: no touch, touch, enwrap, and engulf (also see Figure S1 and Videos [Link], [Link], [Link], [Link]). NeuN^+^TUNEL^+^ cells were frequently observed to be engulfed in Iba1^+^ cells, indicating phagocytosis of apoptotic neurons by microglia/macrophages. Scale bar: 7 μm. E, Percentage of microglia/macrophages showing no touch, touch, enwrap, or engulf relations with neurons in the noninjured contralesional hemisphere (left panel), ipsilesional infarct core and inner peri‐infarct areas (middle panel), and proximal area (right panel) 3‐7 days after dMCAO. F, Quantification of NeuN/TUNEL/Iba1 triple‐positive cells (microglia and macrophages engulfing dead/dying neurons) in the infarct core (area a in B) 3‐7 days after dMCAO. G, Phagocytic index was calculated as the percentage of dead/dying neurons engulfed by microglia/macrophages in the infarct core (area a in B) 3‐7 days after dMCAO. n = 4‐5 mice per group. *P < .05, **P < .01, ***P < .001 between different time points after ischemia, as indicated. ^*P* < .05, ^^*P* < .01, ^^^*P* < .001 vs. noninjured contralateral hemisphere. ns, no significant difference

Our RNA‐seq data strongly suggest a significant role of macrophages in the clearance of dead/dying cells in the poststroke brain. We therefore performed immunofluorescence staining to verify macrophage efferocytosis after dMCAO and characterized its spatial and temporal profiles. NeuN and Iba1 were used to label neurons and myeloid cells in the brain, respectively, 3, 5, and 7 days after dMCAO. Brain infarct was well formed in the ipsilesional hemisphere at this subacute stage after ischemia, in which dead/dying neurons with condensed NeuN immunosignal were frequently observed (Figure 4A). Also present in the infarct core were the Iba1^+^ activated microglia and macrophages characterized by an ameboid morphology. To assess ischemia‐induced changes in the distribution and morphology in neurons and myeloid cells, the total number of neurons (NeuN^+^ cells), microglia/macrophages (Iba1^+^ cells), and NeuN/Iba1 double‐labeled cells were counted in the infarct core (a), inner peri‐infarct area (b), proximal area (c), and distal area (d) (Figure 4B, C) 3‐7 days after dMCAO and in the nonischemic contralateral hemisphere as control. Brain ischemia induced prominent loss of neurons in the infarct core and inner peri‐infarct area 3‐7 days after dMCAO (Figure 4C top panel). In the proximal area, we observed a decrease in NeuN^+^ cells 3 days after dMCAO compared with contralateral controls, which later returned to baseline levels 5‐7 days after dMCAO (Figure 4C top panel). Since we defined the border between the proximal area and inner peri‐infarct area based on the immunofluorescence intensity of NeuN, this fluctuation in NeuN^+^ cell numbers 3‐7 days after dMCAO might reflect the shift of the arbitrarily defined “infarct border” but not the actual change in cell numbers. The number of Iba1^+^ microglia/macrophage increased in the infarct area following ischemia (Figure 4C middle panel), which may result from the infiltration of blood‐derived cells or in situ migration of existing parenchymal myeloid cells. With the accumulation of microglia/macrophages, the numbers of NeuN^+^/Iba1^+^ cells increased 3‐7 days after dMCAO and peaked at 5 days (Figure 4C bottom panel), suggesting that dead/dying cells may be removed by microglia/macrophages. The numbers of NeuN^+^ and Iba1^+^ cells in the distal area remained unchanged 3‐7 days after dMCAO compared to levels in the contralateral controls (Figure 4C).

To further investigate the interactions between neurons and phagocytes in the brain after cerebral ischemia, we classified all Iba1^+^ cells into four categories according to their contact size with NeuN^+^ neurons (see Section 2): no touch, touch, enwrap, and engulf (Figure 4D, Figure S1 and Videos [Link], [Link], [Link], [Link]). Quantification of Iba1^+^ microglia/macrophages under each category revealed distinct distribution patterns of cells in the core/inner peri‐infarct area, proximal area, and the contralateral hemisphere (Figure 4E and Table S1). In the nonischemic contralateral hemisphere, 81.2% of Iba1^+^ cells touched a neuron and 14.5% of Iba1^+^ cells enwrapped a neuron (Figure 4E left panel), consistent with an established role for resting microglia in synaptic pruning in the homeostatic brain.^39^ After dMCAO, the number of “touch” Iba1^+^ cells was significantly less in the infarct core area than contralateral side (Figure 4E middle panel and Table S1), whereas the “enwrap” and “engulf” Iba1^+^ cells increased, suggesting closer interactions between phagocytes and neurons in response to ischemic injury. Compared with the contralateral hemisphere, “engulf” Iba1^+^ cells appeared 3 days after dMCAO (*P* = .076), reached a peak level 5 days after dMCAO (*P* < .001) and remained present for at least 7 days after dMCAO (Table S1). In the proximal area (Figure 4E right panel), “engulf” Iba1^+^ cells were rarely detected up to 7 days after dMCAO, but the “enwrap” ones were much higher than the contralateral hemisphere, perhaps reflecting an intermediate state between the infarct core and uninjured tissue. TUNEL labeling of apoptotic cells revealed that most NeuN^+^ cells engulfed by Iba1^+^ cells were apoptotic neurons. The number of NeuN^+^TUNEL^+^Iba1^+^ cells peaked 5 days after dMCAO (Figure 4F), however, no significant difference was detected in the phagocytic index among the three time points examined (Figure 4G). These findings showed that dead neurons induced by ischemia undergo phagocytic clearance by microglia/macrophage in the brain, with maximal clearance 5 days postischemia, which persisted for at least 7 days after brain ischemia.

### 3.5 PPARγ and STAT6 are predicted to be master regulators of macrophage efferocytosis and inflammation resolution in the poststroke brain

**Figure 5:**
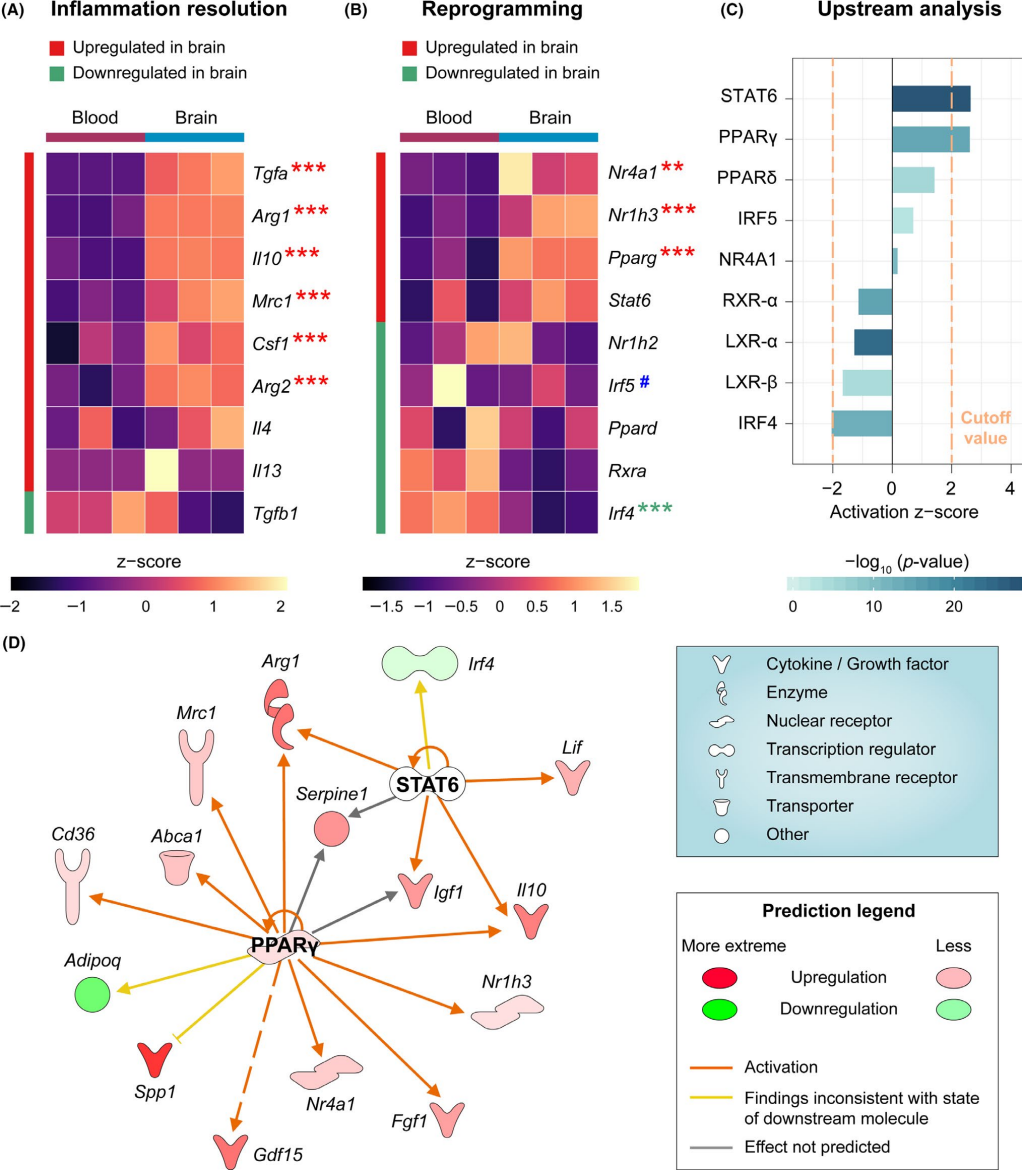
PPARγ and STAT6 are predicted to be master regulators of macrophage efferocytic function in the poststroke brain. A, Expression profiles of a panel of molecules that promote the resolution of inflammation. B, Expression profiles of a panel of transcription regulators, which affect the efferocytic capacity of phagocytes. Asterisks label the significantly upregulated (red) or downregulated (green) genes in brain macrophages, where ** and *** indicate an adjusted *P*‐value <.01 and .001, respectively. # indicates the gene product typically inhibits efferocytic capacity. C, *Upstream Regulator* analyses were performed by IPA on DEGs in macrophages from post‐dMCAO brain versus blood. The activation *z*‐score and *P*‐value of overlap were shown for a panel of molecules that are potential upstream regulators. The cutoff values for predicated activation or inhibition were *z*‐score >2 or <−2 and *P*‐value <.01. PPARγ and STAT6 were predicted to be upstream regulators that were strongly activated in post‐dMCAO brain macrophages. D, DEGs in the brain macrophages that are regulated by PPARγ and STAT6 are shown in a network view, with annotations on the types of their gene products

An important consequence and benefit of efferocytosis is alleviation of local inflammation after dead cells and debris are cleared from the injured tissue. Therefore, we examined the expression levels of a panel of common antiinflammatory and prorepair markers in monocytes/macrophages in the post‐dMCAO brain and blood and found that six of them were significantly upregulated in brain macrophages (Figure 5A), including genes encoding cytokines and growth factors secreted into the extracellular space (*Tgfa*, *Il10*, *Csf1*), and prorepair enzymes in the cytoplasm (*Arg1*, *Arg2*). Given these potential inflammation‐resolving and salutary activities of macrophages, we explored whether there were master transcription regulators that determined the macrophage transcriptome after stroke. We assessed the expression of several transcriptional factors, which were previously reported^40^, ^41^, ^42^, ^43^ to contribute to phagocytosis‐related genomic reprogramming of macrophages in peripheral organs or in vitro cell cultures. In our dataset (Figure 5B), the expression of *Nr4a1*, *Nr1h3,* and *Pparg* was significantly upregulated in brain macrophages, whereas *Irf5*, a negative regulator of efferocytosis,^41^ was downregulated in brain macrophages, although the change was not significant. Since transcription regulators may be activated even in the absence of an increase in expression, we performed IPA to predict the functional status of each regulator, taking into consideration the expression profiles of the entire genome and prior knowledge of expected effects between transcriptional regulators and their target genes.^44^ Among all molecules examined, PPARγ and STAT6 were the only two upstream regulators that were predicted to be strongly activated (*z*‐score >2 and *P* < .01) in brain macrophages (Figure 5C). Representative DEGs downstream of these two regulators (Figure 5D) included antiinflammatory factors and growth factors (eg, IL‐10, Arginase 1, IGF1, LIF, GDF‐15, FGF1), nuclear receptors (eg, LXR‐α, NR4A1), and efferocytic receptors and transporters (eg, CD36, ABCA1). Collectively, these two molecules may play a central role in dictating the proefferocytic transcriptome of brain macrophages after ischemic stroke.

## 4 DISCUSSION

**Figure 6:**
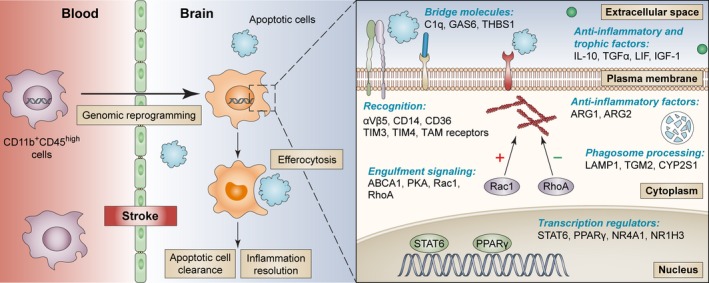
Summary of the role of macrophages in efferocytosis and inflammation resolution in the poststroke brain. Following ischemic stroke, blood monocytes/macrophages (CD11b^+^CD45^high^ cells) invade the postischemic brain and undergo genomic reprogramming that enhances their efferocytic capacity. This genomic reprogramming results in a cascade of changes such as the upregulation of plasma membrane receptors and extracellular bridge molecules that assist with the recognition of apoptotic cells. Intracellular signaling is subsequently triggered, which stimulates cytoskeletal rearrangement, engulfment of apoptotic cells, and phagosome internalization. A group of transcription regulators are activated during macrophage efferocytosis, leading to the production of antiinflammatory and trophic factors. The genes listed were differentially expressed in brain macrophages versus their counterparts in the blood, whose products participate in each of these critical steps of efferocytosis. The efferocytic macrophages play a pivotal role in the clearance of dead cells and resolution of inflammation in the brain, ultimately promoting the neurorestorative processes after stroke

In this study, we used RNA‐seq profiling to elucidate the genomic changes in monocytes/macrophages upon entering the brain after ischemic stroke, and the functional implications of this altered macrophage transcriptome on poststroke brain injury and recovery. Our results reveal genomic adaptations in brain macrophages that drive these cells into a proefferocytic and antiinflammatory phenotype, which facilitate the clearance of dead cells and elicit regenerative responses in the neurovascular niche (Figure 6).

The importance of efferocytosis in the poststroke brain has largely been overlooked; thus, not much is known about the contributing cells and the molecular mechanisms regulating efferocytosis. Efferocytosis is a special type of phagocytosis that has unique characteristics compared with the typical phagocytosis process of microorganisms; the latter process involves the recognition of pathogen‐associated molecular patterns (PAMPs) derived from microorganisms by pattern recognition receptors (PRRs).^45^ In contrast, efferocytosis is initiated by DAMPs released by the injured cell, which engage specialized receptors that recognize the “eat‐me” markers on dead cells. The intracellular signaling pathways downstream of these receptors are also different from those downstream of the PRRs.

Engulfment of dead cells and subsequent processing of phagosomes are the execution steps of efferocytosis, which require the participation of specific cellular machinery and remodeling of the actin cytoskeleton.^36^ We observed upregulation in the expression of several intracellular signaling molecules involved in cellular engulfment in brain macrophages 5 days after dMCAO and concomitant upregulation of many cytoskeletal regulators, implying the involvement of active cytoskeletal remodeling of brain macrophages at this stage after ischemia. However, future studies measuring the protein levels of these and other factors are warranted to further confirm this involvement. In contrast, there was relatively negligible processing of phagosomes in brain macrophages. Since our RNA‐seq data were obtained from a single time point (5 days) after dMCAO, the data may not capture the complete time course of efferocytic activities of brain macrophages. Our in vivo immunohistochemistry data indicated that phagocytosis of apoptotic neurons peaked 5 days after dMCAO. Future studies expanding the time window of examination are warranted to confirm the temporal profile of activation of engulfment signaling and phagosome formation in situ. It should also be noted that although cytoskeletal remodeling is a prerequisite of efferocytosis, the cytoskeletal regulators examined here are nonspecific to engulfment activity and could implicate other biological processes involving cytoskeleton changes. For example, macrophages can repair injured vessels through direct mechanical contact with endothelium,^46^ for which Rac1 activity and cytoskeleton changes are required. Other activities such as cell migration, cell adhesion, and diapedesis also involve cytoskeletal changes.

Using Iba1 to label phagocytes in the brain, we observed active efferocytosis during the subacute stage after dMCAO targeting apoptotic neurons. Since all myeloid‐lineage cells express Iba1, including resident microglia, CNS border‐associated macrophages, and blood‐borne monocytes/macrophages,^47^, ^48^, ^49^ we could not differentiate between the contributions of activated resident microglia and monocyte‐derived macrophages to overall efferocytic activity. On the other hand, the bulk RNA‐seq on brain CD11b^+^CD45^high^ cells used in this study also could not distinguish between resident microglia that upregulate CD45 upon activation and monocyte‐derived macrophages, which have intrinsically high CD45 expression.^50^ Nevertheless, high expression of CD45 represents functionally active myeloid cells in the poststroke brain, and future studies may employ single‐cell RNA‐seq to further differentiate the roles of microglia versus macrophages. Microglia depletion approaches^51^, ^52^ may also shed light on the relative contribution to overall phagocytosis by CNS microglia and monocyte‐derived macrophages.

We identified two potential master regulators, that is, PPARγ and STAT6, which may determine the transcriptome of macrophages and shape their functional status in the poststroke brain. PPARγ was previously reported to promote phagocytic clearance of dead cells by macrophages in in vitro cultures, peripheral organs, and after intracerebral hemorrhage.^53^, ^54^, ^55^ Furthermore, a recent study from our group demonstrated a pivotal role of STAT6 in microglia/macrophage efferocytosis in the brain after ischemic stroke.^56^ In this study, we focused on molecules that are established regulators of phagocytosis. However, the prediction made by upstream regulator analysis may also uncover novel regulatory molecules. Genetic or pharmacologic approaches that target these regulators may be a promising treatment to manipulate efferocytosis to promote inflammation resolution, eventually facilitating functional recovery after stroke.

In summary, we report that brain macrophages harbor a transcriptome favoring efferocytosis during the subacute stage after ischemic stroke and may represent a rational therapeutic target to enhance resolution of inflammation and brain repair. In peripheral organs, efferocytosis is generally believed to be a beneficial process that maintains tissue homeostasis and expedite tissue restoration after injury. Intriguingly, a controversial role of phagocytosis in the poststroke brain has also been proposed, wherein phagocytes may attack viable neurons or oligodendrocyte precursor cells and exacerbate brain injury after cerebral ischemia.^57^, ^58^ In our study, most phagocytosed neurons were TUNEL‐positive apoptotic cells; however, whether phagocytosis is detrimental or salutary in the brain should be assessed considering its overall impact on the functional recovery after stroke. Furthermore, the phagocytotic activity of macrophages may vary at different injury stages after ischemic stroke or be altered by biological variables such as age and sex.^59^, ^60^ These are additional critical factors to consider when developing efferocytosis‐manipulating strategies.

## CONFLICT OF INTEREST

The authors declare no conflict of interest.
