# Supplementary material for: Macrophages reprogram after ischemic stroke and promote efferocytosis and inflammation resolution in the mouse brain
Source: CNS Neurosci Ther. 2019 Nov 7;25(12):1329–42. doi: 10.1111/cns.13256 (PMC6887920; doi:10.1111/cns.13256)
Supplement: Supplementary file 1 [file CNS-25-1329-s001.pdf]

Supporting Information

Supplementary Video 1. 3D remodeling of the "no touch" relationship between microglia/macrophages and neurons by Imaris. Shown are fluorescence signals of Iba1 (green), NeuN (red), and DAPI (blue) taken from the ipsilateral cortex 5 days after dMCAO. There is no contact surface area between NeuN and Iba1 immunofluorescence in the volume examined. A total depth of 21 μm of the tissue was scanned and remodeled.

Supplementary Video 2. 3D remodeling of the "touch" relationship between microglia/macrophages and neurons by Imaris. Shown are fluorescence signals of Iba1 (green), NeuN (red), and DAPI (blue) taken from the contralateral cortex 5 days after dMCAO. The contact surface areas between NeuN and Iba1 immunofluorescence are marked in yellow. A total depth of 21 μm of the tissue was scanned and remodeled.

Supplementary Video 3. 3D remodeling of the "enwrap" relationship between microglia/macrophages and neurons by Imaris. Shown are fluorescence signals of Iba1 (green), NeuN (red), and DAPI (blue) taken from the proximal area of the ipsilesional cortex 5 days after dMCAO. The contact surface areas between NeuN and Iba1 immunofluorescence are marked in yellow. A total depth of 21 μm of the tissue was scanned and remodeled.

Supplementary Video 4. 3D remodeling of the "engulf" relationship between microglia/macrophages and apoptotic neurons by Imaris. Shown are fluorescence signals of Iba1 (green), NeuN (red), TUNEL (white), and DAPI (blue) taken from the infarct core of the ipsilesional cortex 5 days after dMCAO. Apoptotic neurons are double-labeled by TUNEL (white) and NeuN (red). The contact surface areas between NeuN and Iba1 immunofluorescence are marked in yellow. A total depth of 21 μm of the tissue was scanned and remodeled.

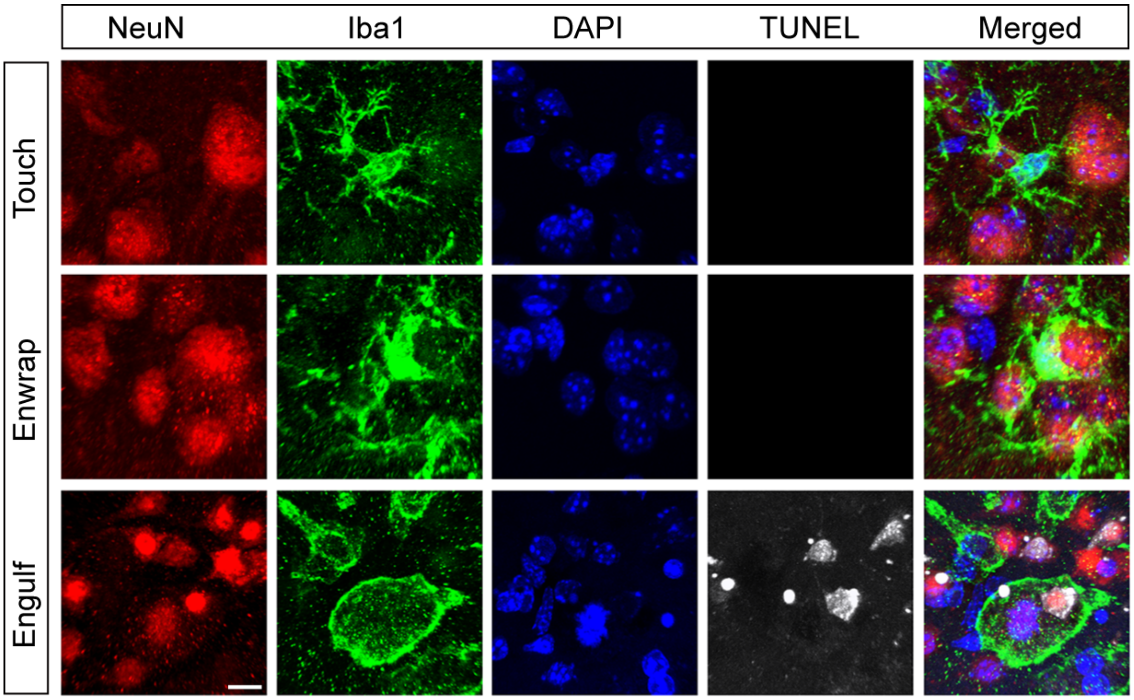

Supplementary Figure 1. Activated microglia and macrophages phagocytose dead/dying neurons at the subacute stage after cerebral ischemia (related to Figure 4D).

Mice were subjected to permanent focal cerebral ischemia induced by dMCAO. Shown are representative images of brain sections stained with NeuN (red), Iba1 (green), DAPI (blue), and TUNEL (white) demonstrating the interactions between neurons and microglia/macrophages 5 days after dMCAO. Color-merged images are shown in the 5th column. Scale bar: 10 µm.

Supplementary Table 1. Statistics reporting

FIGURE	LABEL	GROUP	*p* VALUE
Figure 4C<br>(NeuN+ cells)	^ Infarct core *vs.* contralateral hemisphere at indicated time points<br>^ Proximal area *vs.* contralateral hemisphere at indicated time points<br>^ Peri-infarct area *vs.* contralateral hemisphere at indicated time points<br>* DPI 3 *vs.* DPI 5 in proximal area	Core	Contralateral-DPI 3 $p = 0.0007$ (^^^)<br>Contralateral-DPI 5 $p = 0.0032$ (^^)<br>Contralateral-DPI 7 $p = 0.0006$ (^^^)<br>DPI 3-DPI 5 $p > 0.9999$<br>DPI 3-DPI 7 $p > 0.9999$<br>DPI 5-DPI 7 $p > 0.9999$
		Peri-infarct	Contralateral-DPI 3 $p < 0.0001$ (^^^)<br>Contralateral-DPI 5 $p < 0.0001$ (^^^)<br>Contralateral-DPI 7 $p < 0.0001$ (^^^)<br>DPI 3-DPI 5 $p > 0.9999$<br>DPI 3-DPI 7 $p > 0.9999$<br>DPI 5-DPI 7 $p > 0.9999$
		Proximal	Contralateral-DPI 3 $p = 0.0393$ (^)<br>Contralateral-DPI 5 $p > 0.9999$<br>Contralateral-DPI 7 $p > 0.9999$<br>DPI 3-DPI 5 $p = 0.0086$ (**)<br>DPI 3-DPI 7 $p = 0.4643$<br>DPI 5-DPI 7 $p > 0.9999$
		Distal	Contralateral-DPI 3 $p = 0.4149$<br>Contralateral-DPI 5 $p > 0.9999$<br>Contralateral-DPI 7 $p > 0.9999$<br>DPI 3-DPI 5 $p > 0.9999$<br>DPI 3-DPI 7 $p > 0.9999$<br>DPI 5-DPI 7 $p > 0.9999$
Figure 4C<br>(Iba1+ cells)	^ Infarct core *vs.* contralateral hemisphere at indicated time points<br>^ Proximal area *vs.* contralateral hemisphere at indicated time points<br>^ Peri-infarct area *vs.* contralateral hemisphere at indicated time points<br>* DPI 5 *vs.* DPI 7 in core area<br>* Difference between three time points in peri-infarct area	Core	Contralateral-DPI 3 $p < 0.0001$ (^^^)<br>Contralateral-DPI 5 $p < 0.0001$ (^^^)<br>Contralateral-DPI 7 $p = 0.0342$ (^)<br>DPI 3-DPI 5 $p = 0.1146$<br>DPI 3-DPI 7 $p = 0.4354$<br>DPI 5-DPI 7 $p = 0.0008$ (***)
		Peri-infarct	Contralateral-DPI 3 $p < 0.0001$ (^^^)<br>Contralateral-DPI 5 $p > 0.9999$<br>Contralateral-DPI 7 $p = 0.0048$ (^^)<br>DPI 3-DPI 5 $p < 0.0001$ (***)<br>DPI 3-DPI 7 $p > 0.9999$<br>DPI 5-DPI 7 $p = 0.0040$ (**)
		Proximal	Contralateral-DPI 3 $p > 0.9999$<br>Contralateral-DPI 5 $p = 0.0516$<br>Contralateral-DPI 7 $p < 0.0001$ (^^^)<br>DPI 3-DPI 5 $p = 0.0971$<br>DPI 3-DPI 7 $p < 0.0001$ (***)<br>DPI 5-DPI 7 $p < 0.0001$ (***)
		Distal	Contralateral-DPI 3 $p > 0.9999$<br>Contralateral-DPI 5 $p > 0.9999$<br>Contralateral-DPI 7 $p > 0.9999$<br>DPI 3-DPI 5 $p > 0.9999$<br>DPI 3-DPI 7 $p > 0.9999$<br>DPI 5-DPI 7 $p > 0.9999$

Figure 4C (NeuN+/Iba1+ Cells)	^ Infarct core *vs.* contralateral hemisphere at indicated time points<br>^ Peri-infarct area *vs.* contralateral hemisphere at indicated time points.<br>* Difference between three time points in core area	Core	Contralateral-DPI 3 $p < 0.0001$ (^^^)<br>Contralateral-DPI 5 $p < 0.0001$ (^^^)<br>Contralateral-DPI 7 $p < 0.0001$ (^^^)<br>DPI 3-DPI 5 $p < 0.0001$ (***)<br>DPI 3-DPI 7 $p = 0.0008$ (***)<br>DPI 5-DPI 7 $p < 0.0001$ (***)
		Peri-infarct	Contralateral-DPI 3 $p = 0.0169$ (^)<br>Contralateral-DPI 5 $p > 0.9999$<br>Contralateral-DPI 7 $p = 0.7122$<br>DPI 3-DPI 5 $p = 0.0683$<br>DPI 3-DPI 7 $p > 0.9999$<br>DPI 5-DPI 7 $p > 0.9999$
		Proximal	Contralateral-DPI 3 $p > 0.9999$<br>Contralateral-DPI 5 $p > 0.9999$<br>Contralateral-DPI 7 $p > 0.9999$<br>DPI 3-DPI 5 $p > 0.9999$<br>DPI 3-DPI 7 $p > 0.9999$<br>DPI 5-DPI 7 $p > 0.9999$
		Distal	Contralateral-DPI 3 $p > 0.9999$<br>Contralateral-DPI 5 $p > 0.9999$<br>Contralateral-DPI 7 $p > 0.9999$<br>DPI 3-DPI 5 $p > 0.9999$<br>DPI 3-DPI 7 $p > 0.9999$<br>DPI 5-DPI 7 $p > 0.9999$
Figure 4E (Contralateral)	Not label in figure 4E	DPI 3	Touch-No touch $p < 0.0001$<br>Engulf-Enwrap $p = 0.0660$<br>Enwrap-Touch $p < 0.0001$<br>Enwrap-No touch $p >0.9999$<br>Engulf-Touch $p < 0.0001$<br>Engulf-No touch $p = 0.5776$
		DPI 5	Touch-No touch $p < 0.0001$<br>Engulf-Enwrap $p = 0.1441$<br>Enwrap-Touch $p < 0.0001$<br>Enwrap-No touch $p = 0.2375$<br>Engulf-Touch $p < 0.0001$<br>Engulf-No touch $p > 0.9999$
		DPI 7	Touch-no touch $p < 0.0001$<br>Engulf-Enwrap $p = 0.5763$<br>Enwrap-touch $p < 0.0001$<br>Enwrap-No touch $p = 0.8647$<br>Engulf-Touch $p < 0.0001$<br>Engulf-No touch $p > 0.9999$
Figure 4E (Core & Inner peri-infarct)	* "touch" Iba1+ cells between indicated time points post ischemia<br>* "Engulf" Iba1+ cells between indicated time points post ischemia	Between time points	No touch:<br>DPI 3-DPI 5 $p = 0.3158$<br>DPI 3-DPI 7 $p = 0.6116$<br>DPI 5-DPI 7 $p = 0.9018$<br>Touch:<br>DPI 3-DPI 5 $p = 0.0030$ (**)<br>DPI 3-DPI 7 $p = 0.0474$ (*)<br>DPI 5-DPI 7 $p = 0.6712$<br>Enwrap:<br>DPI 3-DPI 5 $p = 0.3440$

			DPI 3-DPI 7 $p = 0.9908$<br>DPI 5-DPI 7 $p = 0.4588$<br>Engulf:<br>DPI 3-DPI 5 $p = 0.0035$ (**)<br>DPI 3-DPI 7 $p = 0.4283$<br>DPI 5-DPI 7 $p = 0.1253$
	Not label in figure 4E	DPI 3	Touch-No touch $p > 0.9999$<br>Engulf-Enwrap $p > 0.9999$<br>Enwrap-Touch $p > 0.9999$<br>Enwrap-No touch $p > 0.9999$<br>Engulf-Touch $p = 0.7964$<br>Engulf-No touch $p > 0.9999$
		DPI 5	Touch-No touch $p = 0.0026$<br>Engulf-Enwrap $p = 0.0026$<br>Enwrap-Touch $p = 0.6786$<br>Enwrap-No touch $p = 0.2063$<br>Engulf-Touch $p < 0.0001$<br>Engulf-No touch $p = 0.6766$
		DPI 7	Touch-No touch $p = 0.2096$<br>Engulf-Enwrap $p > 0.9999$<br>Enwrap-Touch $p = 0.4973$<br>Enwrap-No touch $p > 0.9999$<br>Engulf-Touch $p = 0.2264$<br>Engulf-No touch $p > 0.9999$
Figure 4E (Proximal)	No significant difference	Between time points	No touch:<br>DPI 3-DPI 5 $p > 0.9999$<br>DPI 3-DPI 7 $p = 0.8225$<br>DPI 5-DPI 7 $p > 0.9999$<br>Touch:<br>DPI 3-DPI 5 $p = 0.5291$<br>DPI 3-DPI 7 $p = 0.1688$<br>DPI 5-DPI 7 $p > 0.9999$<br>Enwrap:<br>DPI 3-DPI 5 $p > 0.9999$<br>DPI 3-DPI 7 $p > 0.9999$<br>DPI 5-DPI 7 $p > 0.9999$<br>Engulf:<br>DPI 3-DPI 5 $p > 0.9999$<br>DPI 3-DPI 7 $p > 0.9999$<br>DPI 5-DPI 7 $p > 0.9999$
	Not label in figure 4E	DPI 3	Touch-No touch $p < 0.0001$<br>Engulf-Enwrap $p = 0.0002$<br>Enwrap-Touch $p = 0.0007$<br>Enwrap-No touch $p = 0.0012$<br>Engulf-Touch $p < 0.0001$<br>Engulf-No touch $p < 0.0001$
		DPI 5	Touch-No touch $p < 0.0001$<br>Engulf-Enwrap $p = 0.0001$<br>Enwrap-Touch $p = 0.0780$<br>Enwrap-No touch $p = 0.0083$<br>Engulf-Touch $p < 0.0001$<br>Engulf-No touch $p > 0.9999$
		DPI 7	Touch-No touch $p = 0.0003$

			Engulf-Enwrap $p = 0.0098$<br>Enwrap-Touch $p = 0.3551$<br>Enwrap-No touch $p = 0.0839$<br>Engulf-Touch $p < 0.0001$<br>Engulf-No touch $p > 0.9999$
Figure 4E (Core& Inner peri-infarct vs. Contralateral)	^ No touch between core & Inner peri-infarct and contralateral<br>^ Touch between core & Inner peri-infarct and contralateral<br>^ Enwrap between core & Inner peri-infarct and contralateral<br>^ Engulf between core & Inner peri-infarct and contralateral	DPI 3	No touch $p = 0.0759$<br>Touch $p < 0.0001$ (^^^)<br>Enwrap $p = 0.6313$<br>Engulf $p = 0.5177$
		DPI 5	No touch $p < 0.0001$ (^^^)<br>Touch $p < 0.0001$ (^^^)<br>Enwrap $p > 0.9999$<br>Engulf $p < 0.0001$ (^^^)
		DPI 7	No touch $p = 0.0012$ (^^)<br>Touch $p < 0.0001$ (^^^)<br>Enwrap $p = 0.1466$<br>Engulf $p = 0.0007$ (^^^)
Figure 4E (Proximal vs. Contralateral)	^ Touch between core & Inner peri-infarct and contralateral<br>^ Enwrap between core & Inner peri-infarct and contralateral	DPI 3	No touch $p > 0.9999$<br>Touch $p = 0.6582$<br>Enwrap $p = 0.1762$<br>Engulf $p > 0.9999$
		DPI 5	No touch $p = 0.1229$<br>Touch $p < 0.0001$ (^^^)<br>Enwrap $p = 0.0001$ (^^^)<br>Engulf $p > 0.9999$
		DPI 7	No touch $p = 0.3701$<br>Touch $p < 0.0001$ (^^^)<br>Enwrap $p = 0.0167$ (^)<br>Engulf $p > 0.9999$
Figure 4F	* *vs*. indicated time point<br>^ *vs*. contralateral	None	Contralateral-DPI3 $p = 0.0032$ (^^)<br>Contralateral-DPI5 $p < 0.0001$ (^^^)<br>Contralateral-DPI7 $p = 0.0528$<br>DPI 3-DPI 5 $p = 0.0122$ (*)<br>DPI 5-DPI 7 $p = 0.0019$ (**)
Figure 4G	^ *vs*. contralateral<br>ns: no significant difference	None	Contralateral-DPI3 $p=0.0088$ (^^)<br>Contralateral-DPI5 $p=0.0005$ (^^^)<br>Contralateral-DPI7 $p=0.0473$ (^)
